# Combined lncRNA and mRNA Expression Profiles Identified the lncRNA–miRNA–mRNA Modules Regulating the Cold Stress Response in *Ammopiptanthus nanus*

**DOI:** 10.3390/ijms24076502

**Published:** 2023-03-30

**Authors:** Ming Zhu, Qianshi Dong, Jie Bing, Songbuerbatu, Lamei Zheng, Tashi Dorjee, Qi Liu, Yijun Zhou, Fei Gao

**Affiliations:** 1 Key Laboratory of Mass Spectrometry Imaging and Metabolomics (Minzu University of China), National Ethnic Affairs Commission, Beijing 100081, China; 2 Key Laboratory of Ecology and Environment in Minority Areas (Minzu University of China), National Ethnic Affairs Commission, Beijing 100081, China; 3 College of Life and Environmental Sciences, Minzu University of China, Beijing 100081, China; 4 College of Life Sciences, Beijing Normal University, Beijing 100080, China

**Keywords:** lncRNA, miRNA, cold stress, *Ammopiptanthus nanus*, regulatory module

## Abstract

Long non-coding RNAs (lncRNAs) have been shown to play critical regulatory roles in plants. *Ammopiptanthus nanus* can survive under severe low-temperature stress, and lncRNAs may play crucial roles in the gene regulation network underlying the cold stress response in *A. nanus*. To investigate the roles of lncRNAs in the cold stress response of *A. nanus*, a combined lncRNA and mRNA expression profiling under cold stress was conducted. Up to 4890 novel lncRNAs were identified in *A. nanus* and 1322 of them were differentially expressed under cold stress, including 543 up-regulated and 779 down-regulated lncRNAs. A total of 421 lncRNAs were found to participate in the cold stress response by forming lncRNA–mRNA modules and regulating the genes encoding the stress-related transcription factors and enzymes in a cis-acting manner. We found that 31 lncRNAs acting as miRNA precursors and 8 lncRNAs acting as endogenous competitive targets of miRNAs participated in the cold stress response by forming lncRNA–miRNA–mRNA regulatory modules. In particular, a cold stress-responsive lncRNA, *TCONS00065739*, which was experimentally proven to be an endogenous competitive target of *miR530*, contributed to the cold stress adaptation by regulating *TZP* in *A. nanus*. These results provide new data for understanding the biological roles of lncRNAs in response to cold stress in plants.

## 1. Introduction

Temperature is one of the most important ecological factors affecting plant growth and geographical distribution, and high- or low-temperature stress has adverse effects on plant growth and development. Cold stress inhibits various biological processes by directly affecting multiple metabolic reactions and produces other stresses, such as osmotic and oxidative stresses, indirectly in plant. Finally, cold stress negatively affects the production and quality of crops and other economically important plants. The molecular mechanism in the cold stress response is one of the hotspots in plant science, and a number of studies have investigated the cold response in plants using physiological, transcriptomic, and proteomic approaches [1,2,3]. A batch of stress-related genes were identified and further analyzed functionally via transgenic studies [4], and several cold signal perception and responsive pathways, such as the ICE1–CBF–COR pathway, were elucidated. These studies made substantial contributions to our understanding of cold tolerance mechanisms in plants.

Long non-coding RNAs are functional non-translated molecules greater than 200 nt. They are transcribed by RNA polymerase II (PolII), PolIII, and PolV, and exert their functions through multiple regulatory pathways [5]. Long non-coding RNAs can be divided into six categories according to their genomic location: intergenic lncRNAs, bidirectional lncRNAs, intronic lncRNAs, antisense lncRNAs, sense lncRNAs, and others [6]. Long non-coding RNAs can regulate protein-coding genes through various ways such as epigenetic regulation, transcriptional regulation, post-transcriptional regulation, and protein activity regulation [7]. Long non-coding RNAs can perform their biological functions in three ways, including regulating the expression of coding genes in cis or trans [8], indirectly regulating the expression of coding genes as precursors of miRNAs [9], and indirectly regulating proteins by acting as the mimetic targets of miRNAs [10].

Unlike the lncRNAs in mammals that have gained extensive attention in recent years, only a few studies have reported the roles of lncRNAs in plants. Recently, some studies found that lncRNAs play important regulatory roles in response to biotic and abiotic stresses in plants and crop models, including *Oryza sativa* [11], *Gossypium hirsutum* [12], and *Arabidopsis thaliana* [13]. In rice, the lncRNA *TCONS_00021861* was shown to inhibit *miR528-3p*-mediated cleavage of *YUCCA7* as a competitor for *YUCCA7*, which increased the resistance of the plants to drought stress [14]. The overexpression of cotton *lncRNA973* improved the salt tolerance in *Arabidopsis*, indicating that *lncRNA973* played a regulatory role in the plant’s response to salt tolerance [15]. In *Arabidopsis*, the lncRNA *asHSFB2a* affected the response of the plants to heat stress by inhibiting the expression of *HSFB2a* [16], and a novel lncRNA, *COLD INDUCED lncRNA 1 (CIL1)*, was identified as a positive regulator of the plant’s response to cold stress [17]. With the rapid development of high-throughput sequencing, the systematic identification of lncRNAs and the expression profiling of lncRNAs under abiotic stress have been performed in many species, especially plant and crop models, which are generally sensitive to abiotic stress [18,19,20]. Considering that some stress-tolerant plants that have lived under multiple stress conditions for a long time may have evolved some gene expression regulation mechanisms to effectively cope with various stress conditions, the study of lncRNAs in stress-tolerant plants can not only expand the repository of plant lncRNAs, but also enrich the understanding of the biological functions of plant lncRNAs.

*Ammopiptanthus nanus* is an evergreen broad-leaved shrub of the genus *Ammopiptanthus* in the leguminous family, mainly distributed in Wuqia County, Xinjiang Uygur Autonomous Region, China and Kyrgyzstan [21]. *Ammopiptanthus nanus* is a relic of the Tertiary period and can grow in an environment with multiple abiotic stresses [22,23,24]. Previous studies revealed that *A. nanus* showed high levels of tolerance to drought, high salinity, high temperature, cold and freezing stresses, and was used as an important material for studying the stress tolerance mechanism of woody plants [25]. The chromosome-level reference genome of *A. nanus* has been reported and researchers have carried out a number of studies, using physiological and biochemical methods, transcriptomics, proteomics, and other omics techniques to analyze the abiotic stress tolerance mechanism and identify stress tolerance-related genes in *A. nanus* [26,27,28,29,30]. A recent study revealed that conserved and lineage-specific miRNAs contributed to the cold stress response by regulating ROS homeostasis and stress signaling by negatively regulating the corresponding targets [31]. Long non-coding RNAs may play important roles in the cold stress response in *A. nanus* by regulating mRNA/protein-coding genes. In the present paper, lncRNAs and their targets were systematically identified in *A. nanus* using high-throughput sequencing technology, and the lncRNA–miRNA–mRNA and lncRNA–mRNA modules regulating the cold stress response in *A. nanus* were identified via the integrative analysis of lncRNA and mRNA. This study provides new insight into the lncRNA-mediated gene regulation network associated with the cold stress response in *A. nanus*.

## 2. Results

### 2.1. Physiological and Biochemical Indexes of A. nanus Leaves under Cold Stress

**Figure 1:**
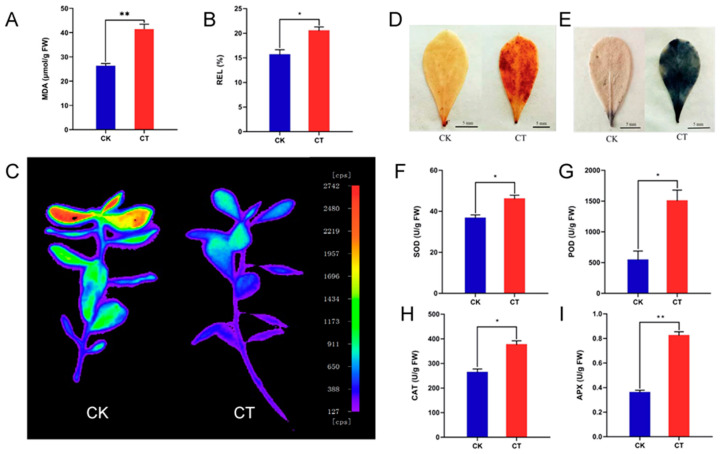
The physiological and biochemical indexes of *A. nanus* seedlings under cold stress. (**A**) The content of MDA. (**B**) REL values. (**C**) Delayed fluorescence images of the CK and CT. (**D**) DAB staining shows the site of H_2_O_2_ accumulation in leaves under cold stress. The darker the color, the more H_2_O_2_ is accumulated. (**E**) NBT staining shows the site of O^2−^ accumulation in *A. nanus* leaves under cold stress. The darker the blue color, the more O^2−^ is accumulated. (**F**) The enzyme activities of SOD. (**G**) The enzyme activities of POD. (**H**) The enzyme activities of CAT. (**I**) The enzyme activities of APX. Each experiment was performed in three independent biological replicates. Values are expressed as means ± SD (*n* = 3), and the statistically significant differences were evaluated using Student’s *t*-test, * *p* < 0.05, ** *p* < 0.01.

To investigate the effects of cold stress on *A. nanus*, delayed fluorescence imaging, and 3,3′-diaminobenzidine (DAB) and nitroblue tetrazolium (NBT) staining assays were conducted. Malondialdehyde (MDA), Relative electrolyte leakage (REL), and the activities of several antioxidant enzymes were measured in the unstressed seedlings (CK) and the cold-stressed seedlings (CT). The values of MDA and REL were increased in the CT, indicating that cold stress caused damage to the leaf cells. Compared to the control group, the fluorescence intensity of the CT was weaker as revealed in the delayed fluorescence imaging (Figure 1A,B), indicating that photosynthesis was inhibited to some extent by the cold stress treatment (Figure 1C). The increased intensity of DAB and NBT staining in the CT demonstrated that more reactive oxygen species (ROS) were accumulated in the leaves after the cold stress treatment (Figure 1D,E). The raised level of superoxide dismutase (SOD), peroxidase (POD), catalase (CAT), and ascorbate peroxidase (APX) activities suggested that the antioxidant enzymes were activated in the *A. nanus* leaves under cold stress (Figure 1B,F–H). In brief, the cold stress treatment caused damage to the plant cells, inhibited photosynthesis, promoted ROS accumulation, and activated the antioxidant system in the leaves of *A. nanus*.

### 2.2. Genome-Wide Identification of lncRNAs in A. nanus

To systematically identify lncRNAs in the *A. nanus* leaves, strand-specific RNA-seq libraries from the leaves of cold-stressed and unstressed *A. nanus* seedlings were constructed and sequenced in three biological replicates. The high-throughput sequencing generated more than 494 million raw reads, with no less than 74.45 million raw reads from each library. More than 97.48% of the bases had quality scores > Q20, indicating that the quality of the RNA-seq data was highly credible. After filtering the low-quality reads and rRNAs, approximately 48 million clean reads were obtained (Table 1).

The clean reads were further mapped onto the *A. nanus* genome using TopHat 2(2.1.1) [32], and the transcripts were then assembled and annotated using the Cufflinks package [33]. Known mRNAs were identified according to the *A. nanus* genome annotation [34], and this led to the identification of more than 28,000 unique mRNAs from the six cDNA libraries (Table 2). Many new transcript isoforms were also generated, and ultimately, a total of 45,788 unique transcripts were assembled from the high-throughput RNA-Seq data from the cold-stressed group and control group.

**Figure 2:**
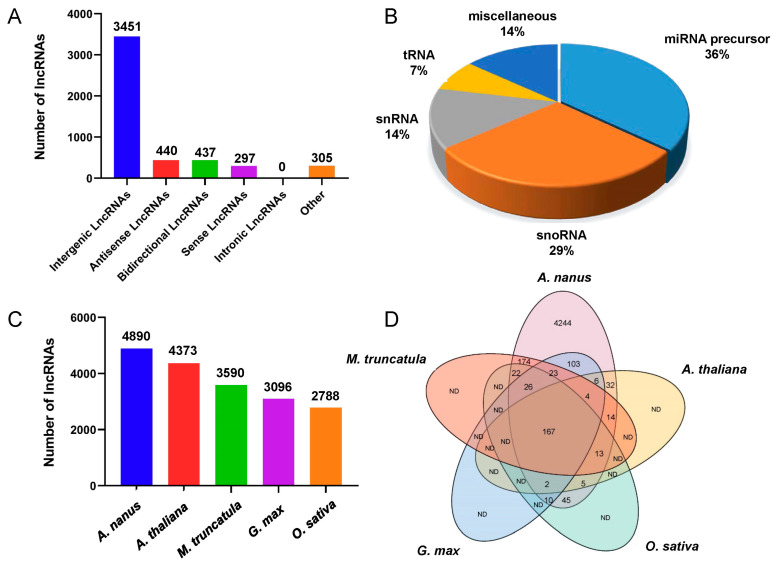
Identification of lncRNAs in *A. nanus*. (**A**) Classification of the lncRNAs identified in *A. nanus* according to the locations in the genome. (**B**) Housekeeping ncRNA and miRNA precursors predicted from the six libraries. (**C**) Number of lncRNAs identified from *A. nanus*, *G. max*, *M. truncatula*, *A. thaliana*, and *O. sativa.* (**D**) The Venn diagram analysis of lncRNAs identified from *A. nanus* with those identified from *G. max*, *M. truncatula*, *A. thaliana*, and *O. sativa*. ND, the number of lncRNAs were not determined.

Finally, we identified 4012, 4078, 3998, 3803, 3784, and 4069 unique lncRNAs from the six cDNA libraries, respectively (Table 2), representing 4890 unique lncRNAs. This number was comparable to that of the lncRNAs in *Arabidopsis* and maize. According to the locations of the lncRNAs in the *A. nanus* genome, all identified *A. nanus* lncRNAs were classified into six categories: intergenic lncRNAs, intronic lncRNAs, bidirectional lncRNAs, antisense lncRNAs, sense overlapping lncRNAs, and other lncRNAs. Of the six categories, the intergenic lncRNA category was the most abundant, accounting for 70.57% of the total lncRNAs (Figure 2). No intronic lncRNAs were found in the *A. nanus* leaves.

Housekeeping ncRNAs, such as tRNAs, snRNAs, and snoRNAs, and miRNA precursors are two categories of lncRNAs that function differently from other lncRNAs. To identify these conserved ncRNAs, the identified lncRNAs were further aligned to the Rfam database. A total of 147 known ncRNAs were found, including 53 miRNA precursors, 42 snoRNAs, 20 snRNAs, and 11 tRNAs (Table S1). To identify other homologous lncRNAs, the remaining lncRNAs were aligned with the lncRNAs from *A. thaliana*, *Oryza sativa*, *Glycine max*, and *Medicago truncatula* in the CANTATAdb 2.0 database [http://cantata.amu.edu.pl/ (accessed on 16 August 2022)] using the BioEdit program [35]. As expected, 444 lncRNA orthologs were found in *M. truncatula*, 342 lncRNA orthologs were found in *G. max*, and 244 lncRNA orthologs were detected in *A. thaliana*, while only 290 homologous lncRNAs were present in *O. sativa*. (Figure 2C and Table S2). A total of 4244 *A. nanus* lncRNAs (86.79% of the total number of *A. nanus* lncRNAs) did not show apparent similarities to any lncRNAs in *G. max*, *M. truncatula*, *A. thaliana*, and *O. sativa*. Only 167 *A. nanus* lncRNAs (3.42%) existed in all of the four plant species, supporting the opinion that lncRNAs are far less conservative than protein-coding RNAs.

### 2.3. Target Prediction for lncRNAs Acting in Cis in A. nanus

**Figure 3:**
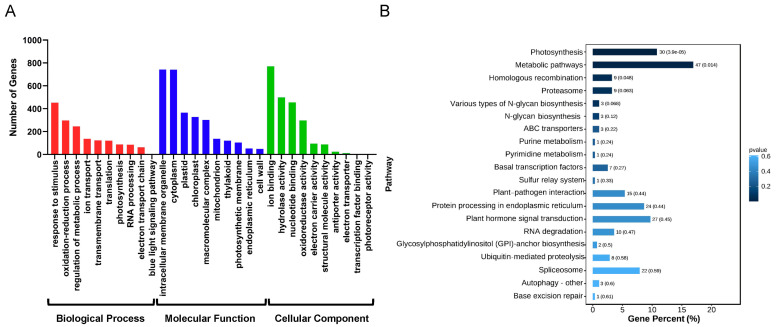
Functional analysis of cis-regulated lncRNA–mRNA pairs. (**A**) GO term classification of targets. The *x*-axis indicates GO terms, and *y*-axis indicates the number of genes. The red bar charts represent the biological process, the blue bar charts indicate the molecular function, and the green bar charts represent the cellular component. (**B**) KEGG pathways analysis of targets (top 20). The *x*-axis represents the percentage of genes, and the *y*-axis indicates the pathways.

Long non-coding RNA can affect the transcription of nearby protein-coding genes. In *A. nanus*, a total of 3423 lncRNAs were found to potentially act in cis on 4728 protein-coding genes and formed 6211 cis-regulated lncRNA–mRNA pairs (Table S3). The GO enrichment analysis of these targets revealed the potential roles of the cis-acting lncRNAs in the growth, development, and stress response in *A. nanus* (Figure 3A). These targets were assigned to 221 GO biological process categories, 41 cellular component categories, and 114 molecular function categories. The top three enriched biological process terms were the oxidation-reduction process (GO: 0055114), regulation of metabolic process (GO: 0019222), and response to chemical (GO: 0042221). For the cellular component, intracellular membrane-bounded organelle (GO: 0043231), cytoplasm (GO: 0005737), and plastid (GO: 0009536) were the three most enriched GO terms. For the molecular function, ion binding (GO: 0043167), hydrolase activity (GO: 0016787), and nucleotide binding (GO: 0000166) were the top three enriched terms. The KEGG enrichment analysis showed that 37 pathways might be affected by these cis-acting lncRNAs, including photosynthesis, metabolic pathways, and homologous recombination (Figure 3B).

### 2.4. Ammopiptanthus nanus lncRNAs Regulate a Variety of Biological Processes by Affecting miRNAs

Long non-coding RNAs may function as the precursors or the targets of miRNAs to participate in the gene regulation network. First, by aligning the miRNA precursors to the *A. nanus* lncRNAs, 53 lncRNAs were found to be the precursors of the miRNAs, and 100 miRNAs, which belonged to 29 miRNA families, were probably generated from these precursors. Second, using psRNAtarget, 127 lncRNAs were found to be the putative targets of 88 conserved miRNAs in *A. nanus* (Table S4).

**Figure 4:**
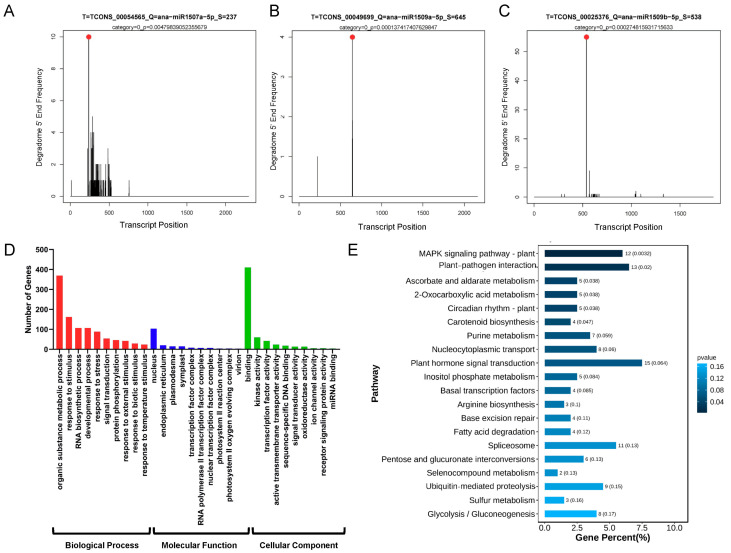
T-plots of target lncRNAs of the miRNAs and functional analysis of target mRNAs of the miRNAs affected by lncRNAs. (**A**) T-plots of *ana-miR1507a-5p-TCONS00054565*. The red dot indicates the cleavage site. (**B**) T-plots of *ana-miR1509a-5p-TCONS00049699*. The red dot indicates the cleavage site. (**C**) T-plots of *ana-miR1509b-5p-TCONS00025376*. The red dot indicates the cleavage site. (**D**) GO term classification of targets. The *x*-axis indicates GO terms, and *y*-axis indicates the number of genes. The red bar charts represent the biological process, the blue bar charts indicate the molecular function, and the green bar charts represent the cellular component. (**E**) KEGG pathways analysis of targets (top 20). The *x*-axis represents the percentage of genes, and the *y*-axis indicates the pathways.

To experimentally identify the target genes in the predicted miRNAs in *A. nanus* in batch, a degradome sequencing dataset was reanalyzed using CleaveLand pipeline with the *A. nanus* lncRNAs as the transcripts, and 13 lncRNAs were identified as the targets (categories ≤ 4) of the *A. nanus* miRNAs (Table S5). Of these, three lncRNAs, *TCONS00054565* targeted by *ana-miR1507a-5p*, *TCONS00049699* targeted by *ana-miR1509a-5p*, and *TCONS00025376* targeted by *ana-miR1509b-5p*, were targets of the highest credibility (Figure 4A–C).

To understand the biological function of the lncRNAs, the targets of the miRNAs affected by the lncRNAs were predicted bioinformatically and GO and KEGG enrichment analyses were performed for these targets. A total of 2764 miRNA-target pairs (representing 1192 unique mRNAs) were predicted. Using GO enrichment analysis, the targets were assigned to 434 biological process categories, 29 cellular component categories, and 325 molecular function categories (Figure 4D). The top three enriched biological process terms were: containing organic metabolic substance process (GO: 0071704), response to stimulus (GO: 0050896), and gene expression (GO: 0010467). For the cellular fraction, nucleus (GO: 0005634), endoplasmic reticulum (GO: 0005783), and plasmodesma (GO: 0009506) were the three most represented GO terms. For the molecular function, binding (GO: 0005488), kinase activity (GO: 0016301), and transcription factor activity (GO: 0003700) were the top three enriched terms. The KEGG enrichment analysis of these targets revealed 94 enriched pathways, including the MAPK signaling pathway, plant–pathogen interaction, and ascorbate and aldarate metabolism (Figure 4E).

### 2.5. Responses of lncRNAs to Cold Stress in A. nanus

**Figure 5:**
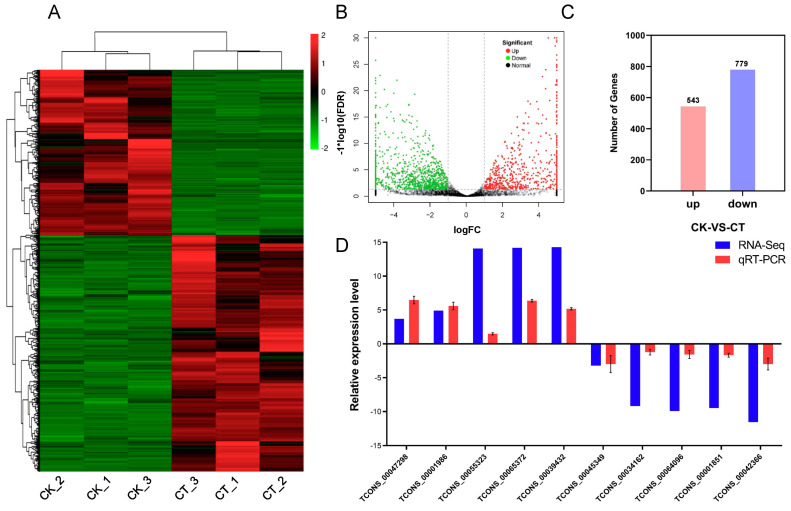
Differentially expressed lncRNAs under cold stress in *A. nanus*. (**A**) Heatmap of differentially expressed lncRNAs. (**B**) Volcano plot of differentially expressed lncRNAs. (**C**) Number of differentially expressed lncRNAs with up-regulated or down-regulated change patterns. (**D**) qRT-PCR verifies the change patterns in lncRNAs under cold stress. The blue bar graph represents the relative expression level in lncRNA sequencing, and the red bar graph represents the result of qRT-PCR. Each experiment was performed in three independent biological replicates, and the error bar shows the SD.

By comparing the abundance of each lncRNA in the CK and CT, a total of 1322 differentially expressed lncRNAs were identified, including 543 up-regulated and 779 down-regulated lncRNAs (Figure 5A–C). To confirm the differential expression of the lncRNAs calculated using the RNA-seq data, 10 lncRNAs were randomly selected for qRT-PCR analysis. The expression pattern revealed using qRT-PCR for each lncRNA was similar to that of the RNA-seq, indicating that the differentially expressed lncRNAs identified using the RNA-seq data were reliable (Figure 5D).

### 2.6. Response of mRNAs to Cold Stress in A. nanus

**Figure 6:**
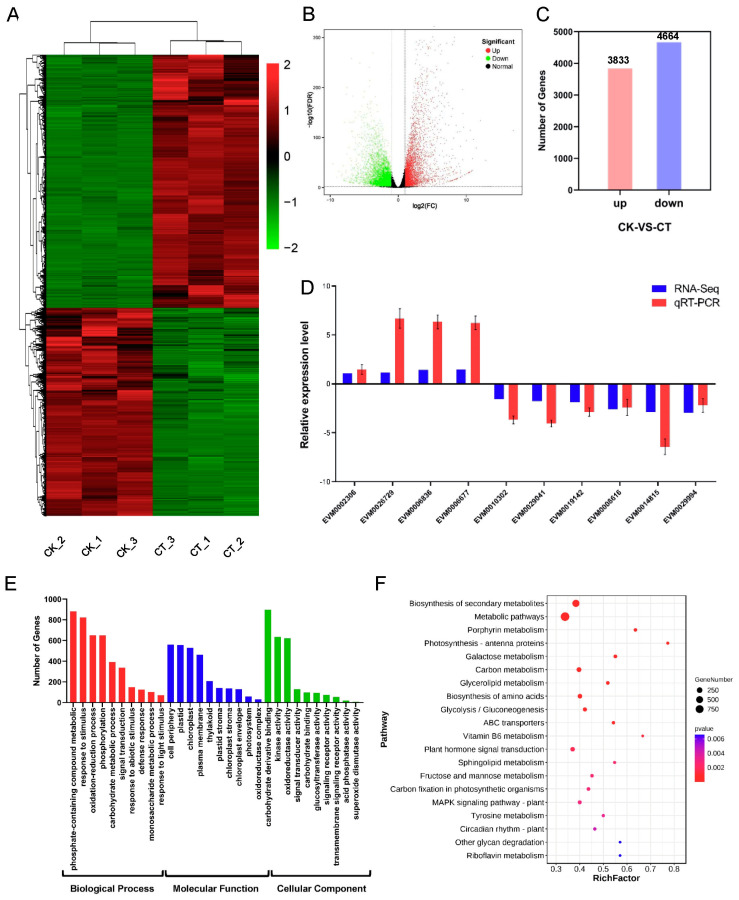
The differentially expressed mRNAs under cold stress in *A. nanus*. (**A**) Heatmap of differentially expressed mRNAs. (**B**) Volcano plot of differentially expressed mRNAs. (**C**) Number of differentially expressed mRNAs with up-regulated or down-regulated change patterns. (**D**) qRT-PCR verifies the change patterns of mRNAs under cold stress. The blue bar graph represents the relative expression level of mRNA sequencing, and the red bar graph represents the result of qRT-PCR. Each experiment was performed in three independent biological replicates, and the error bar shows SD. (**E**) GO enrichment analysis. (**F**) KEGG pathway enrichment analysis. The *x*-axis represents the rich factor, and the *y*-axis indicates the enriched KEGG pathways.

The differential gene analysis revealed that 8497 mRNAs were differentially expressed in *A. nanus* under cold stress, including 3833 up-regulated and 4664 down-regulated mRNAs (Figure 6A–C). To confirm the differential expression of the mRNAs revealed using the RNA-seq analysis, 10 mRNAs were randomly selected for qRT-PCR. The expression pattern of the mRNAs revealed using qRT-PCR showed the same trend as that of the RNA-seq data (Figure 6D), indicating that the differentially expressed mRNAs identified using RNA-seq were credible.

To reveal the biological process and pathways represented in the 8497 differentially expressed mRNAs, GO and KEGG enrichment analyses were conducted. The top three enriched GO biological process terms were: phosphate-containing compound metabolic (GO: 0006796), response to stimulus (GO: 0050896), and oxidation-reduction process (GO: 0055114). For the cellular component, cell periphery (GO: 0071944), plastid (GO: 0009536), and chloroplast (GO: 0009507) were the three most enriched terms. For the molecular function, carbohydrate derivative binding (GO: 0097367), kinase activity (GO: 0016301), and oxidoreductase activity (GO: 0016491) were the three overrepresented terms (Figure 6E). A total of 133 KEGG pathways were annotated from the 8497 differentially expressed mRNAs, and the enriched pathways included biosynthesis of secondary metabolites, photosynthesis-antenna biosynthesis proteins, circadian rhythm, aminoacyl-tRNA, and plant signal transduction (Figure 6F).

### 2.7. lncRNAs Participate in Cold Stress Response through Regulating mRNAs in Cis

**Figure 7:**
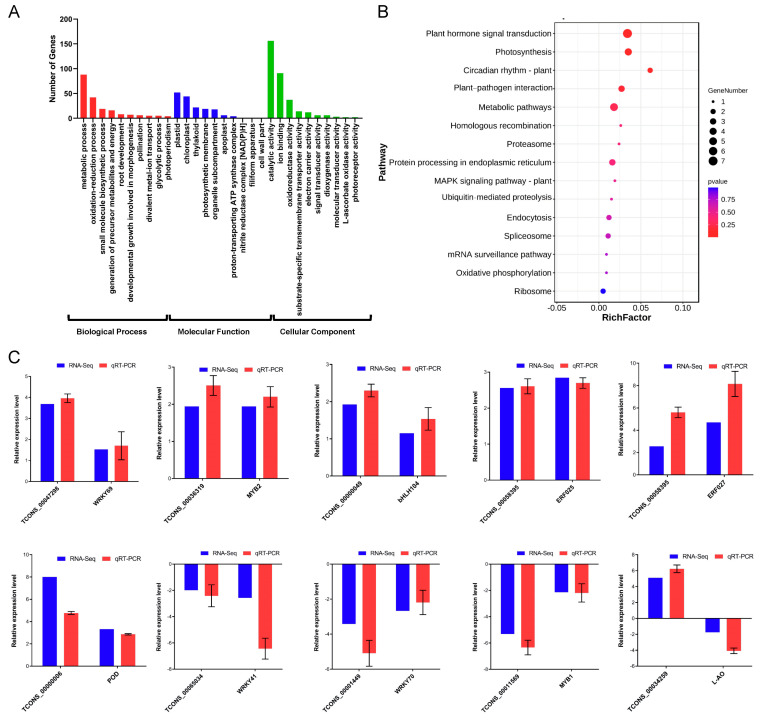
Functional analysis of cis-regulated lncRNA–mRNA pairs related to cold response. (**A**) GO classification of mRNAs. The *x*-axis indicates GO terms, and *y*-axis indicates the number of genes. The red bar charts represent the biological process, the blue bar charts indicate the molecular function, and the green bar charts represent the cellular component. (**B**) KEGG pathway analysis of mRNAs (top 20). The *x*-axis represents the percentage of genes, and the *y*-axis indicates the pathways. (**C**) qRT-PCR verifies the expression patterns of 10 cis-regulated lncRNA–mRNA pairs under cold stress.

To understand the regulatory function of the *A. nanus* lncRNAs in the cold stress response, a combined analysis of lnRNA and mRNA was performed, based on the expression data of differentially expressed lncRNAs and differentially expressed mRNAs. A total of 540 cis-regulated pairs were involved in the cold stress response in *A. nanus*, accounting for 8.69% of all cis-regulated pairs identified in *A. nanus* (Table S6). The 540 cis-regulated pairs were composed of 421 differentially expressed lncRNAs and 452 differentially expressed mRNAs. To reveal the biological process and pathways represented in the 452 differentially expressed mRNAs, GO and KEGG enrichment analyses were conducted. The top three enriched GO biological process terms were: metabolic process (GO: 0044710), oxidation-reduction process (GO: 0055114), and small molecule biosynthetic process (GO: 0044283). For the cellular component, plastid (GO: 0009536), chloroplast (GO: 0009507), and thylakoid (GO: 0009579) were the three most enriched terms. For the molecular function, catalytic activity (GO: 0003824), ion binding (GO: 0043167), and oxidoreductase activity (GO: 0016491) were the three overrepresented terms (Figure 7A). The KEGG analysis revealed that plant hormone signal transduction, photosynthesis, and circadian rhythm-plant pathways were regulated in the leaves of *A. nanus* under cold stress (Figure 7B).

The expression pattern of 399 targets in the lncRNAs acting in cis, accounting for 73.89% of all lncRNAs acting in cis, were positively correlated with that of the corresponding lncRNAs. Nine of these cis-regulated lncRNA–mRNA pairs were further validated using qRT-PCR analysis, including *TCONS00047298-WRKY69 (EVM0026729)*, *TCONS00036319-MYB309 (EVM0015717)*, *TCONS00000049-bHLH104 (EVM0009916)*, *TCONS00058395-ERF025 (EVM0026054)*, *TCONS00058395-ERF027 (EVM0035043)*, *TCONS00000006-POD (EVM0001176)*, *TCONS00065034-WRKY41 (EVM0014815)*, *TCONS00001449-WRKY70 (EVM0029994)*, and *TCONS00011569-MYB308 (EVM0025673)*. The expression pattern of a small proportion of cis-regulated pairs were negatively correlated (26.11%, 141/540), including *TCONS00034259-L-AO (EVM0023147)*, which was validated using qRT-PCR (Figure 7C).

### 2.8. Cold Stress-Responsive lncRNAs Representing miRNA Precursors

**Figure 8:**
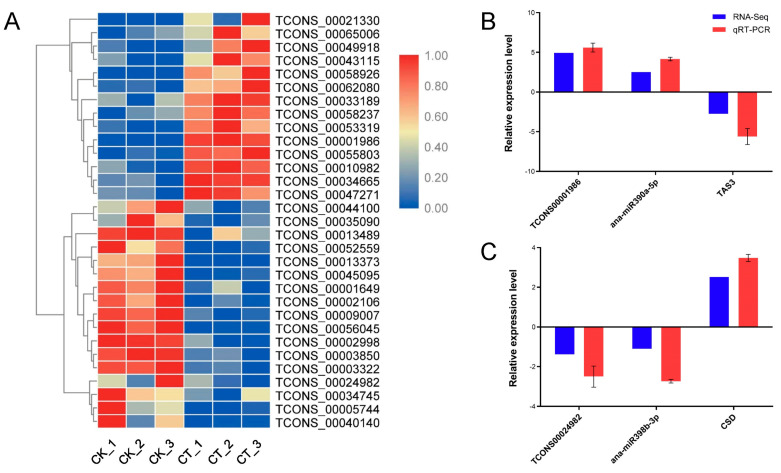
Differential expression of the cold stress-responsive lncRNAs representing miRNA precursors. (**A**) Heatmap of the lncRNAs representing miRNA precursors related to cold stress in *A. nanus*. (**B**) qRT-PCR verifies the expression patterns of *TCONS00001986*, *miR390*, and *TAS3*. (**C**) qRT-PCR verifies the expression patterns of *TCONS00024982*, *miR398*, and *CSD*.

By comparing the abundance of each lncRNA in the libraries from the CK and CT, a total of 31 lncRNAs that represented miRNA precursors were found to be responsive to cold stress, with 14 up-regulated and 17 down-regulated lncRNAs (Figure 8A and Table S7). The derived miRNAs belonged to 20 miRNA families. The combined analysis of our lncRNA and mRNA expression profiles, and previously reported miRNA expression data suggested that two lncRNAs, TCONS00001986 and TCONS00024982, were involved in cold stress by negatively regulating TAS3 and CSD, the targets of miR390 and miR398, respectively, affecting miR390 and miR398. Further qRT-PCR analysis validated these results (Figure 8B,C).

### 2.9. lncRNA–miRNA–mRNA Networks Construction and Analysis

**Figure 9:**
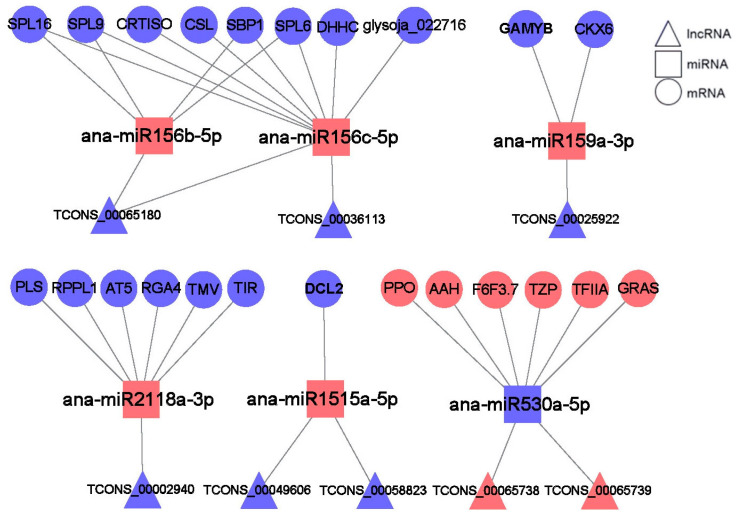
lncRNA–miRNA–mRNA networks involved in the cold stress response in *A. nanus*. lncRNAs are represented by triangles, miRNAs by squares, and mRNAs by circles. Red represents up-regulated expression pattern and blue represents down-regulated pattern.

lncRNAs can bind to miRNAs, thereby affecting the negative regulation of the miRNAs on their protein-coding target genes. Based on the targeting relationship and expression profiles of the lncRNAs, mRNAs, and miRNAs, several lncRNA–miRNA–mRNA networks that might regulate the cold stress response in *A. nanus* were constructed. These networks contained 8 lncRNAs, 6 miRNAs, and 23 mRNAs (Figure 9 and Table S8). The majority of the miRNAs in these networks were up-regulated under cold stress, including *miR156b/c*, *miR159a*, *miR2118a*, and *miR1515a*, and the corresponding targets were down-regulated. Only one miRNA, *miR530a*, was down-regulated under cold stress, and the targeted lncRNAs (*TCONS00065738* and *TCONS00065739*) and mRNAs were up-regulated.

### 2.10. miR530 Targets the lncR5739 (TCONS00065739) and the TZP

**Figure 10:**
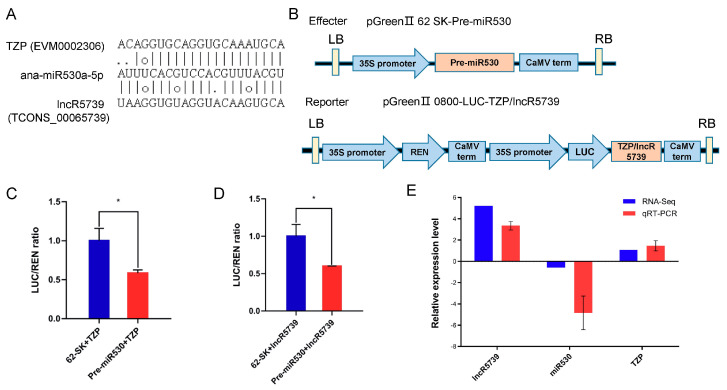
Dual-luciferase reporter assay demonstrates that the *lncR5739* and *TZP* gene is targeted by *miR530*. (**A**) Binding sites of *miR530* on *lncR5739* and *TZP* as predicted by psRNA target. (**B**) Dual-luciferase reporter vector construction. (**C**) The ratio of LUC/REN in the 62 SK + TZP and pre-miR530 + TZP cells. (**D**) The ratio of LUC/REN in the 62 SK + lncR5739 and pre-miR530 + lncR5739 cells. (**E**) Values are expressed as means ± SD (*n* = 3), and the statistically significant differences were evaluated using Student’s *t*-test, * *p* < 0.05. qRT-PCR analysis of the *lncR5739*, *TZP*, and *miR530*. The *y*-axis shows the relative expression with *U6* or actin as the internal reference. Data are averaged over three technical replicates.

Compared with other conservative miRNAs such as miR156, there are relatively few studies on miR530. Thus, we further analyzed the function of the lncRNA–miRNA– mRNA network formed by miR530. Using the online software TAPIR, the *lncR5739* (*TCONS00065739*) and the *TZP* gene were predicted to be the target of *miR530* (Figure 10A). To experimentally verify the targeting relationship of *miR530* to the *lncR5739* and the *TZP* gene, a dual-luciferase reporter assay was performed. Compared with 62 SK + TZP, the LUC/REN ratio was significantly decreased in the pre-miR530 + TZP cells, indicating that miR530 negatively regulated the expression of *TZP* (Figure 10B). At the same time, compared with 62 SK + lncR5739, the LUC/REN ratio was significantly decreased in the pre-miR530 + lncR5739 cells (Figure 10C), indicating that miR530 negatively regulated the expression of *lncR5739* (Figure 10D). The reverse change pattern between *miR530* and *TZP/lncR5739* in cold-stressed *A. nanus* revealed using the RNA-seq and qRT-PCR analyses also validated the target relationship of *miR530*, *TZP*, and *lncR5739* (Figure 10E).

### 2.11. Overexpression of lncR5739 Enhanced the Cold Stress Tolerance in Tobacco

**Figure 11:**
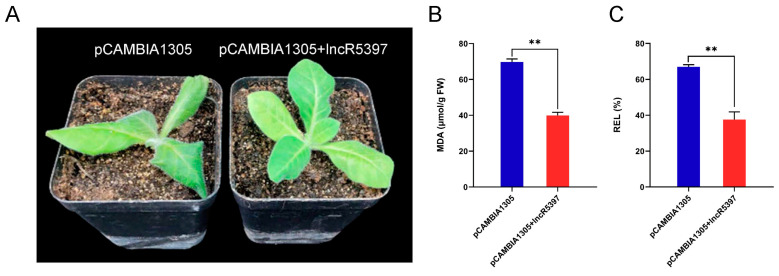
Phenotype analysis of transient overexpression of *lncR5739* in tobacco infiltrated by *Agrobacterium tumefaciens*. (**A**) The growth state of the control and tobacco plants overexpressing *lncR5739*. (**B**) The content of MDA. (**C**) REL values. Values are expressed as means ± SD (*n* = 3), and the statistically significant differences were evaluated using Student’s *t*-test: ** *p* < 0.01.

To investigate the effect of *lncR5739* on the cold stress response in *A. nanus*, *Agrobacterium tumefaciens* expressing pCAMBIA1305-GFP was used to overexpress the *lncR5739* gene in tobacco leaves. The phenotypic differences in the tobacco plants were observed after cold treatment for 24 h (−4 °C), and two biochemical indexes, MDA and REL, were measured. Compared with the control, the tobacco plants overexpressing *lncR5739* showed a better growth state (Figure 11A). The values of MDA and REL were significantly lower in the leaves of the tobacco plants overexpressing *lncR5739* than in the control plants (Figure 11B,C), indicating that *lncR5739* might play a positive role in regulating the response of the plants to cold stress.

## 3. Discussion

*Ammopiptanthus nanus* is a rare, evergreen broad-leaved shrub that grows in central Asia, and this plant species has a strong tolerance to low temperatures. Analyzing the regulation mechanism of gene expression in *A. nanus* in response to low-temperature stress will help to improve the understanding of how the plants adapt to seasonal low temperatures in the autumn and winter. Two previous studies investigated the gene differential expression associated with the low-temperature stress response in the genus *Ammopiptanthus* [36,37], and a recent study identified cold stress-responsive miRNAs and discussed the putative biological roles of these miRNAs in *A. nanus* [31]. However, how the lncRNAs play roles in the regulatory network of gene expression related to the low-temperature response has not been cleared. In the present study, combined lncRNA and mRNA expression profiling analyses were conducted to understand how lncRNAs regulated gene expression in response to cold stress.

Cold stress caused significant physiological change to the *A. nanus* seedlings. The MDA and REL measurements indicated that cold stress caused membrane damage to leaf cells in *A. nanus*, and the NBT and DAB staining showed that the ROS levels were increased in the leaves of *A. nanus* under cold stress. The determination of antioxidant enzyme activity demonstrated that some antioxidant enzymes were activated in *A. nanus* under cold stress.

The response of plants to cold stress is a complicated process that is expected to integrate multiple biological processes and metabolic pathways [38]. In line with this view, the GO enrichment analysis of the 8497 differentially expressed mRNAs in *A. nanus* under cold stress revealed that the biological processes including response to stimulus, oxidation-reduction process, signal transduction, and protein phosphorylation played important roles in cold stress response in *A. nanus*. The KEGG enrichment analysis revealed that photosynthesis, photosynthesis-antenna proteins, carbon fixation in photosynthetic organisms, circadian rhythm, peroxisome, plant hormone signal transduction, starch and sucrose metabolism, and carbon metabolism were involved in the response to cold stress in *A. nanus*. Our results were consistent with those of some previous studies, in which photosynthesis was inhibited and the ROS removal system was activated in cold stressed plants [38].

In the present study, the systematic identification of lncRNAs in *A. nanus* was conducted, and a total of 1322 differentially expressed lncRNAs were identified. These differentially expressed lncRNAs might play regulatory roles in the response to cold stress in *A. nanus*. Long non-coding RNAs can achieve biological functions in multiple ways, including directly regulating the expression of protein-coding genes in cis and indirectly regulating the expression of coding genes by affecting miRNA expression [39,40]. In our study, a total of 6211 cis-regulated lncRNA–mRNA pairs, which were composed of 3423 lncRNAs and 4728 mRNAs, were identified in *A. nanus*. By comparing the expression data of the differentially expressed lncRNAs and the differentially expressed mRNAs under cold stress, 540 cis-regulated lncRNA–mRNA pairs, which were composed of 421 differentially expressed lncRNAs and 452 differentially expressed mRNAs, were identified. Of the 452 mRNAs, there were many stress-related transcription factors and enzymes, including mRNA-encoding transcription factors such as *WRKY*, *MYB*, *ERF*, *bHLH*, and mRNAs encoding enzymes including *POD* and *L-AO*. It was speculated that these cis-regulated lncRNA–mRNA pairs played important roles in the response to cold stress in *A. nanus* by regulating the stress-related transcription factors and enzymes.

Generally, there are two ways in which lncRNAs act on miRNAs: one as the precursors of the miRNAs and the other as the targets of the miRNAs. Long non-coding RNAs were found to act as miRNA precursors in response to stress in a variety of plants [41]. In *A. nanus*, 53 lncRNAs were identified as miRNA precursors, and the corresponding miRNAs belonged to 20 miRNA families, including MIR156, MIR159, MIR167, MIR172, MIR390, MIR393, MIR396, and MIR398. It was reported that cold-responsive miRNAs, including *miR156* [42], *miR159* [43], *miR167* [44], *miR172* [45], *miR390* [46], *miR393* [47], *miR396* [48], and *miR398* [49], played critical regulatory roles in the response of plants to cold stress by negatively regulating their targets. Long non-coding RNAs can also act on protein-coding genes that are targeted by miRNAs by competitively binding to the miRNAs, thereby forming lncRNA–miRNA–mRNA regulatory modules [50]. We found that 127 lncRNAs, 1042 mRNAs, and 88 miRNAs could form lncRNA–miRNA–mRNA regulatory modules in *A. nanus*. Based on the targeting relationship of the lncRNAs, mRNAs, and miRNAs, and their expression profiles under cold stress, several lncRNA–miRNA–mRNA modules involved in the gene regulatory network were identified, which were composed of 8 lncRNAs, 6 miRNAs, and 23 mRNAs.

**Figure 12:**
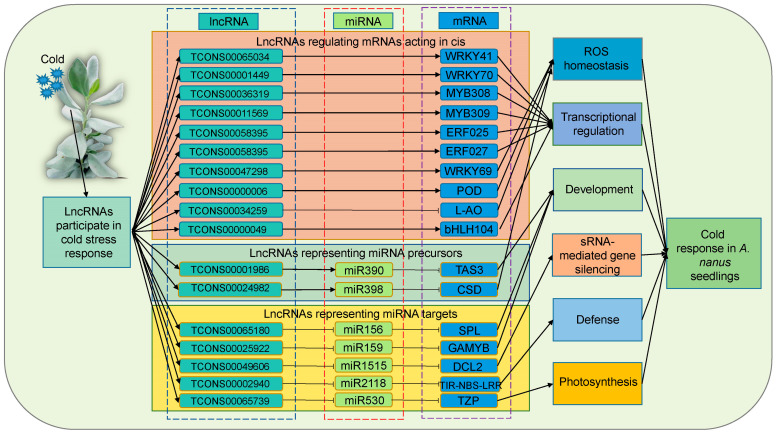
The lncRNA–miRNA–mRNA regulatory network in response to cold in *A. nanus* leaves.

Together, the GO and KEGG analyses of all mRNAs regulated by lncRNAs revealed that *A. nanus* lncRNAs played regulatory roles in the cold stress response by modulation of the biological processes including ROS homeostasis, transcriptional regulation, development, sRNA-mediated gene silencing, defense, and photosynthesis (Figure 12).

Low-temperature stress leads to high levels of ROS accumulation in plant cells, and high levels of ROS cause severe damage to proteins, membrane lipids, and other cellular components. Plants can activate antioxidant enzymes such as *CSD* and *POD* to scavenge excess ROS [51,52]. In the present study, the expression of *TCONS00024982*, a precursor of *miR398*, was decreased under cold stress, while the expression of *miR398* also was decreased. The expression of *CSD*, a target of *miR398*, was increased, and the activity of *CSD* increased, indicating that *TCONS00024982* might down-regulate the expression of *miR398*, which in turn enhanced the expression of *CSD*. The expression of *TCONS00000006*, a lncRNA that targets the *POD* gene, increased under cold stress, while its target, *POD*, also increased, indicating that *TCONS00000006* might up-regulate the expression of *POD* through cis action. These two lncRNAs might be involved in the cold response in *A. nanus* by participating in the maintenance of ROS balance.

Upon perception of a stress signal, the transcription factors are activated by signal transduction pathways to regulate the transcription of cold-responsive genes, and the stress-responsive transcription factors play important roles in the gene regulation network associated with cold stress response in plants [2]. Multiple transcription factors were found to be involved in the response to cold stress, including *WRKY* [53], *MYB* [54], *ERF* [55], and *bHLH* [56]. In our study, the expression of *WRKY69*, *MYB308*, *bHLH104*, *ERF025*, and *ERF027* was increased under cold stress, and *WRKY41*, *WRKY70*, and *MYB309* was decreased. Based on the integrated analysis of the miRNA and lncRNA data, it was found that these differentially expressed cold stress-related transcription factors were regulated by lncRNAs, *TCONS00047298*, *TCONS00011569*, *TCONS00000049*, *TCONS00058395*, *TCONS00001449*, *TCONS00065034*, and *TCONS00036319*. We speculated that these lncRNAs participated in the gene expression network underlying the cold stress response in *A. nanus* by forming cis-regulated pairs with their corresponding targeted transcription factors, i.e., *TCONS00047298-WRKY69*, *TCONS00036319-MYB308*, *TCONS00000049-bHLH104*, *TCONS00058395-ERF025*, *TCONS00058395-ERF027*, *TCONS00001449-WRKY41*, *TCONS00065034-WRKY70*, and *TCONS00011569-MYB309*.

In the photosynthetic system, photopigments (phyA) emit red (R) and far-red (FR) light, while decreases in the ambient temperature and sunshine length in autumn are accompanied by prolongation of the twilight period and decreases in the R: FR ratio, resulting in plants adapting to the cold before the onset of winter [57]. *TZP* was found to positively regulate the phyA signaling required for phyA photoreceptor phosphorylation and was a signaling component closely related to the action of the phytochrome photoreceptors [58]. In *A. nanus*, the cold-responsive *lncR5739* could competitively bind and decrease *miR530* abundance to increase the expression level of *TZP*, a target gene of *miR530*. The dual-luciferase experiments verified the targeting relationship between *miR530* and *lncR5739* and *TZP*, and their expression patterns revealed using qRT-PCR analysis also supported this result. We speculated that *lncR5739* contributes to the cold stress response by reducing available *miR530*, increasing *TZP* abundance, and finally, positively regulating phyA signaling.

## 4. Materials and Methods

### 4.1. Plant Materials and Cold Stress Treatment

*A. nanus* seeds were collected from Wuqia County, Xinjiang Uygur Autonomous Region, China. The seeds of *A. nanus* were soaked in 50 °C water for half an hour. Then, the seeds were sown in vermiculite and per-lite (1:1, *w*/*w*) in a greenhouse at approximately 25 °C and 35% relative humidity with a photo-period of 16 h light and 8 h dark. Eight weeks after germination, a total of 18 *A. nanus* seedlings with similar growth were randomly divided into two groups. One group (unstressed seedlings, CK) was kept in the greenhouse for 7 days at 25 °C, and the other group (cold stress treatment seedlings, CT) was exposed to cold stress treatment (5 °C/4 °C, light/dark). The fully extended leaves on the upper part of the seedlings were collected for RNA extraction and determination of physiological and biochemical indexes.

### 4.2. Delayed Fluorescence Imaging, Physiological and Biochemical Analysis of A. nanus Seedlings

The delayed fluorescence of the aerial parts of *A. nanus* was detected using the NightSHADE LB 985 (Berthold, Bad Wildbad, Germany), and the parameters were set according to the method described in Gould [59]. The accumulation of H_2_O_2_ and O^2−^ in leaves was analyzed using DAB staining and NBT staining (, according to the previously described methods [60]. The REL was measured using the method described by Korhonen [61]. The content of MDA, the activities of POD, SOD, CAT, and APX were measured using kits manufactured by Jiancheng Bioengineering Ltd. (Nanjing, China).

### 4.3. lncRNA and Transcriptome Sequencing

Total RNA samples were extracted using the TRIzol reagent. Approximately 5 μg of total RNA was used to construct ssRNA-seq libraries according to the protocol of Ribo-Zero™ Magnetic Kit (Illumina, San Diego, CA, USA). Paired-end sequencing (150 bp) was conducted on an Illumina Hiseq4000 platform (Illumina, San Diego, CA, USA) at the Gene Denovo (Guangzhou, China), following the manufacturer’s instructions. Three independent biological replicates were set for each group. The raw data of RNA-seq were submitted to the NCBI SRA database with accession numbers SRR11087599, SRR11087600, SRR11087601, SRR11087602, SRR11087603, and SRR11087604.

### 4.4. lncRNA Identification

*A. nanus* reference genome and gene annotation were downloaded from GigaBase [34]. Clean data reads were aligned to the *A. nanus* genome using the TopHat 2.0 program [62] and transcripts were reconstructed using Cufflinks 2.2.1 to obtain known transcripts and novel transcripts. Candidate lncRNAs were considered after removing transcript overlapping with known protein-coding genes, transcripts with FPKM < 0.5, and transcripts with a length shorter than 200 bp. Then, two software programs, Coding Potential Calculator 2.0 (CPC) [63] and Coding-Non-Coding Index v2 (CNCI) [64], were used to evaluate the coding potential of the resulting candidate lncRNAs. The candidate lncRNAs with both CPC and CNCI scores less than 0 were identified as lncRNAs.

### 4.5. Differential Expression Analysis of lncRNAs and mRNAs, and GO and KEGG Pathway Analysis

The read counts from CK and CT were normalized to FPKM [65], and the differentially expressed lncRNAs or mRNAs were determined by comparing the lncRNA and mRNA expression between two groups. Genes with a fold change ≥2 or ≤0.5 and a *p* value < 0.01 were identified as differentially expressed lncRNAs or mRNAs. Heatmaps were generated using TBtools v1.09866 [66]. GO annotations and KEGG pathway analysis were performed using GOseq R package software [67] and the KOBAS v2.0 tool [68].

### 4.6. qRT-PCR Analysis of lncRNAs, miRNAs and mRNAs

RNA samples from leaves of the CK and CT seedlings were prepared using the TRIzol reagent. qRT-PCR analyses of lncRNAs, miRNAs, and mRNAs were performed using the methods described previously [31]. The primers were designed based on the sequences of selected lncRNAs, miRNAs, and mRNAs (Table S9). The internal control genes for lncRNAs and mRNAs were 18S rRNAs, and U6 snRNAs for miRNA. The 2^−ΔΔCt^ method was used to calculate the expression level in lncRNA, miRNA, and mRNA [69], and three biological replicates were performed for each reaction.

### 4.7. Dual-Luciferase Reporter Assay

Dual-luciferase reporter assay was performed according to a previously described method [70]. The precursor sequence of *A. nanus miR530* was ligated to pGreen II 62 SK vector and the sequences of *lncR5739* and *TZP* were ligated to pGreen II 0800-LUC vector. The primer sequences for *pre-miR530*, *lncR5739*, *TZP*, pGreen II 62SK, and pGreen II 0800-LUC were synthesized by Sangon Biotech (Shanghai, China) (Table S9). The luciferase signals were measured using the Vazyme Bio-Lite TM Luciferase Assay system kit (Nanjing, China). Three independent repeats were conducted for this assay.

### 4.8. The Transient Expression of lncRNA in Nicotiana tabacum

The *Agrobacterium*-mediated transient transformation was performed according to a previously described method [71]. The sequence of *A. nanus lncR5739* was ligated to pCAMBIA1305 vector, and then transferred into the *Agrobacterium tumefaciens* strain GV3101. Resuspension, containing 10 mM MES, 10 mM MgCl_2_, and 200 μM acetosyringone, was used to dilute GV3101 bacterial solution to OD 600 = 0.6–0.8. The diluted bacterial suspension penetrated into the leaves of tobacco at the 5–6 leaf stage. After cultivation in darkness for 24 h and then under normal illumination for 48 h, the injected tobacco plants were treated with low temperature stress (−4 °C) for 24 h in an incubator. Then, the injected tobacco leaves were used for cold stress tolerance evaluation and MDA and REL measurements.

## Figures and Tables

**Table 1:** Summary of the sequencing reads.

Library	Raw Reads Number	Clean Reads Number after Filtering Low-Quality Reads (%)	Clean Reads Obtained after Filtering rRNA (%)
CK_1	85,744,166	84,218,844 (98.22%)	84,174,080 (99.95%)
CK_2	91,203,058	89,512,572 (98.15%)	89,370,830 (99.84%)
CK_3	81,083,996	79,176,154 (97.65%)	79,090,710 (99.89%)
CT_1	83,086,322	81,076,694 (97.58%)	80,973,362 (99.87%)
CT_2	78,783,216	76,828,286 (97.52%)	76,785,718 (99.94%)
CT_3	74,450,226	73,215,036 (98.34%)	73,179,680 (99.95%)

**Table 2:** Statistics of the lncRNAs identified from the six libraries.

Library Name	Known mRNA Number	New mRNA Number	Total mRNA Number	New lncRNA Number
CK_1	28,216	12,494	40,710	4012
CK_2	28,373	12,621	40,994	4078
CK_3	27,920	12,506	40,426	3998
CT_1	26,958	12,080	39,038	3803
CT_2	26,741	12,007	38,748	3784
CT_3	28,132	12,307	40,439	4069

## Data Availability

The raw data were deposited in GenBank under six consecutive accession numbers: SRR11087599, SRR11087600, SRR11087601, SRR11087602, SRR11087603, and SRR11087604.

## Supplementary Materials

The following supporting information can be downloaded at: https://www.mdpi.com/article/10.3390/ijms24076502/s1.
